# Differential Responses of Net Ecosystem Exchange of Carbon Dioxide to Light and Temperature between Spring and Neap Tides in Subtropical Mangrove Forests

**DOI:** 10.1155/2014/943697

**Published:** 2014-07-08

**Authors:** Qing Li, Weizhi Lu, Hui Chen, Yiqi Luo, Guanghui Lin

**Affiliations:** ^1^Ministry of Education Key Laboratory for Earth System Modeling, Center for Earth System Science, Tsinghua University, Beijing 100084, China; ^2^Division of Ocean Science and Technology, Graduate School at Shenzhen, Tsinghua University, Shenzhen 518055, China; ^3^College of the Environment and Ecology, Xiamen University, Xiamen, Fujian 361005, China; ^4^Department of Botany and Microbiology, University of Oklahoma, Norman, OK 73019, USA

## Abstract

The eddy flux data with field records of tidal water inundation depths of the year 2010 from two mangroves forests in southern China were analyzed to investigate the tidal effect on mangrove carbon cycle. We compared the net ecosystem exchange (NEE) and its responses to light and temperature, respectively, between spring tide and neap tide inundation periods. For the most time of the year 2010, higher daytime NEE values were found during spring tides than during neap tides at both study sites. Regression analysis of daytime NEE to photosynthetically active radiation (PAR) using the Landsberg model showed increased sensitivity of NEE to PAR with higher maximum photosynthetic rate during spring tides than neap tides. In contrast, the light compensation points acquired from the regression function of the Landsberg model were smaller during spring tides than neap tides in most months. The dependence of nighttime NEE on soil temperature was lower under spring tide than under neap tides. All these results above indicated that ecosystem carbon uptake rates of mangrove forests were strengthened, while ecosystem respirations were inhibited during spring tides in comparison with those during neap tides, which needs to be considered in modeling mangrove ecosystem carbon cycle under future sea level rise scenarios.

## 1. Introduction

Although the importance of mangrove to global carbon cycle is well recognized [1–3], the processes and mechanisms controlling carbon exchange between mangrove forests and atmosphere and its response to tidal inundation are still poorly understood. Many previous researches of tidal effect on mangrove carbon cycle focused mainly on the dissolved inorganic carbon (DIC), dissolved organic carbon (DOC), and particle organic carbon (POC) exchange between coast and sea [4–7]. So far, the control experiments investigating the tidal effect on the photosynthesis and respiration process of mangrove were largely done at individual level [8–11]; thus, it is quite difficult if not impossible to generate common mechanisms for large scale ecosystem CO_2_ exchange under different tidal inundation conditions. Eddy covariance technique provided an ideal tool for monitoring ecosystem level carbon exchange continuously over a long period [12], and in recent years there have been intensive reports on mangrove forests [13–16] and other coastal wetland ecosystems such as salt marshes [17, 18].

Previous analyses of eddy flux data in coastal wetlands revealed that tidal inundation significantly suppressed ecosystem respiration in spring tides comparing to neap tides [18, 19]. However, whether periodic tidal inundation also affects carbon assimilation is still not clear. In mangroves, periodic tidal inundation is one of the most conspicuous features when compared to other terrestrial ecosystems. Tides bring in nutrients and seawater to mangroves, as well as exporting waste pollutants, which has been considered as an auxiliary energy subsidy for mangrove forests to store or export newly fixed carbon [20]. Thus, we can predict that tidal inundation, as one of the most important environmental drivers for coastal wetlands, will have profound impact on mangrove ecosystem carbon balance not only by altering respiration but also by changing carbon assimilation [14, 21]. Furthermore, mangrove forests are regarded to be most susceptible to global change including sea level rise and land-use change due to their residence in the coastal line where the ecosystems are often affected by complex environmental factors from ocean, land, and atmosphere [1]; thus, better understanding of possible relationships between tidal inundation and mangrove CO_2_ exchange is critical for developing models for simulating mangrove future dynamics under various scenarios of sea level rise or coastal land-use [14].

Generally, the tides could be divided into spring tide and neap tide inundation periods. Spring tides happen approximately twice a month when the sun, moon, and earth form a line and the tidal force, while neap tides appear when the sun and moon are separated by 90° [22]. The changes of the tidal inundation depth and periods caused by spring tides and neap tides provide us with an opportunity to compare the responses of mangrove ecosystem CO_2_ exchange to environmental drivers under different patterns of tidal inundation [23]. Two eddy flux towers in mangrove forests were established in southern China in late 2008 or early 2009 to monitor vertical net ecosystem carbon or water exchange. Surface water level and other environmental parameters including net radiation, photosynthesis active radiation, air temperature and humidity, and sediments temperature were also recorded concurrently. In this study, we combined eddy flux data with field records of tidal water inundation depth in two mangrove forests in southern China to analyse the diurnal dynamics of mangrove NEE between spring tide and neap tide inundation periods and its response to light and temperature. We tried to test the hypothesis that the ecosystem respiration of mangrove forests were suppressed more during spring tides than neap tides, which was proposed in a similar research in salt marsh by Guo et al. (2009) [18].

## 2. Materials and Methods

### 2.1. Description of Study Sites

**Figure 1:**
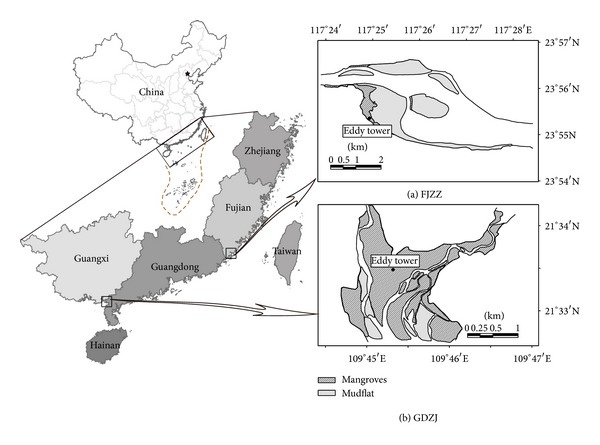
Map for the locations of two study sites of mangrove forests in southern China (FJZZ: Zhangjiangkou Mangrove National Nature Reserve near the Zhangzhou city of Fujian province; GDZJ: Zhanjiang Mangrove National Nature Reserve near the Zhanjiang city of Guangdong province).

Two eddy flux towers were separately located in Zhangjiangkou Mangrove National Nature Reserve near the Zhangzhou city of Fujian province (FJZZ, 23°55′N, 117°23′E) and Zhanjiang Mangrove National Nature Reserve near the Zhanjiang city of Guangdong province (GDZJ, 21°34′N, 109°45′E) in China (Figure 1). All necessary permits for the described field studies were obtained from the Zhangjiangkou Mangrove National Nature Reserve Administration and Zhanjiang Mangrove National Nature Reserve Administration, respectively. And the field studies did not involve any endangered or protected species. The mangrove forests at FJZZ were mainly composed of* Kandelia obovata*,* Avicennia marina, *and* Aegiceras corniculatum*, while the dominant species at GDZJ were* Aegiceras corniculatum*,* Bruguiera gymnorrhiza, and K. obovata*. The average tree height at FJZZ was about 3 m and the average leaf area index (LAI) was 1.7 m^2^ m^−2^. At GDZJ, the average mangrove tree height was 4 m, and the LAI was much higher at 3.8 m^2^ m^−2^.

**Figure 2:**
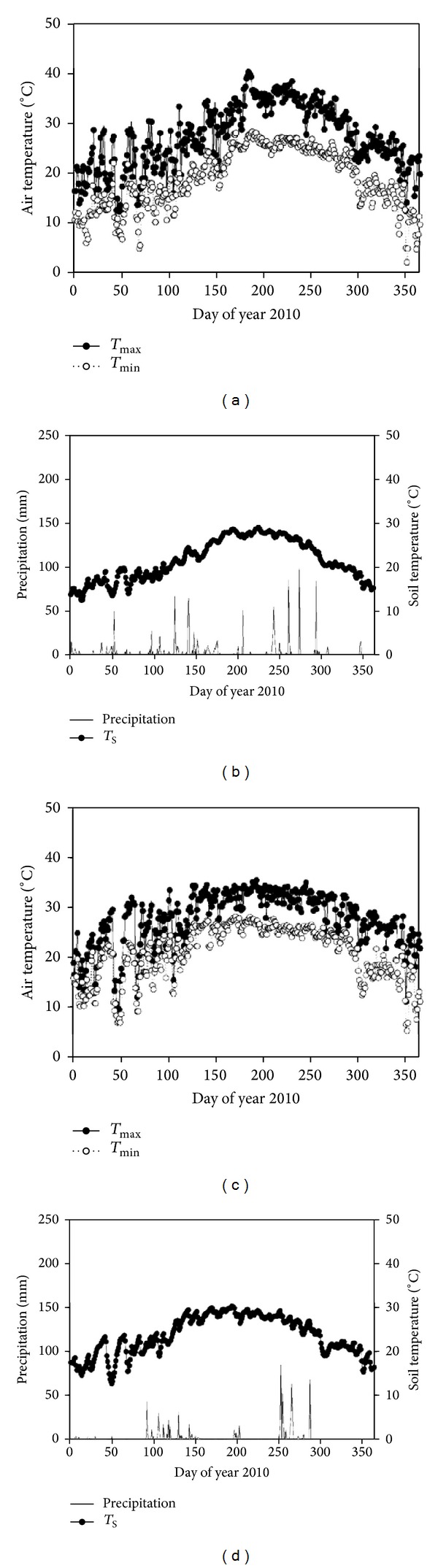
Seasonal variations of major meteorological variables in 2010 for FJZZ and GDZJ.

The annual precipitation was separately 1195 mm and 739 mm, while the air temperature ranged from 10 to 40°C and from 10 to 35°C, respectively, in FJZZ and GDZJ, in 2010 (Figure 2). At FJZZ, the mangrove forest experiences semidiurnal tides and is usually inundated twice during a 24 h period. High tides can reach up to 1.05 m above the sediment surface. However, the sediment surface can be exposed for several days at a time during the annual minima in the solar tidal cycle. At GDZJ, however, the tide belongs to a typical diurnal tide, so the mangrove forest is inundated only once during at most a 24 h period. High tides can reach up to 190 cm above the sediment surface. The contrasting tidal regimes between these two study sites provide a great opportunity to generalize common mechanisms for governing mangrove ecosystem responses to tidal inundation and future sea level rise.

### 2.2. Eddy Flux System and Meteorological Instruments

The eddy flux towers were built in FJZZ and GDZJ according to the standards of AmeriFlux. NEE and sensible and latent heat were measured by the eddy covariance method with a three-axis sonic anemometer (CSAT-3, Campbell Scientific, Inc., Logan, Utah, USA) and open path infrared gas analyzer (IRGA, Li7500, Li-Cor Inc., Lincoln, NE, USA), which were mounted 6 m and 8.6 m above the ground separately in FJZZ and GDZJ. Meteorological parameters were measured with an array of sensors. Net radiation was measured with a four-component net radiometer (CNRI, Kipp and Zonen, Delft, Holland) positioned 12 m above the ground. Photosynthetically active radiation (PAR) was measured at 4 m above the ground using a Li190SB (Li-Cor Inc., Lincoln, NE, USA). Relative humidity and air temperature were measured with shielded sensors (HMP-45C, Vaisala, Helsinki, Finland) at heights of 3 m and 7 m at FJZZ and at heights of 3 m, 7 m, and 16 m at GDZJ. Precipitation amount was recorded with a tipping bucket rain gauge (TE525MM, Texas Electronics, Texas, USA) mounted 12 m above the ground. Soil temperature of 0.10 m and 0.20 m depth was measured at each site using thermistors (model 107, CSI, Campbell Scientific, Inc.). The surface water depths were recorded using YSI level scout (YSI, Yellow Springs, Ohio, USA). Thirty-minute averages were calculated for all micrometeorological measurements and surface water depths for use in further analysis.

### 2.3. EC Measurement

Eddy covariance method has already been proved to be a valuable direct measurement of net carbon exchange between mangrove ecosystem and atmosphere [19]. The following equation presents the calculation of CO_2_ flux [24]:

(1) $$\begin{array}{c} \text{NEE}=\overset{-}{\rho_{\alpha }}\cdot \overset{-}{\omega^{\prime }c^{\prime }}, \end{array}$$

where *ρ*_*α*_ is density of the air and *ω*′ and *c*′ were vertical wind speed and CO_2_ concentration fluctuations from the means, respectively. The over bar in the equation indicated a time average and the primes indicate fluctuations of the mean. Negative CO_2_ flux represents uptake by vegetation and positive flux represents CO_2_ transfer into the atmosphere. Continuous measurement data of the year 2010 in both the two sites were used in our analysis. EC data were collected at 10 Hz on a CR1000 datalogger (Campbell Scientific, Inc., USA) and stored on 2 GB compact flash cards. The collected data were then computed online every 30 min and recorded by the datalogger.

### 2.4. EC Data Processing and Quality Control

Raw EC data were processed with EdiRe program developed by the Institute of Atmospheric and Environmental Sciences, School of GeoSciences, The University of Edinburgh, England. Before calculating the fluxes of CO_2_ and sensible and latent heat, we rotated wind velocity components so that the 30 min mean vertical and cross-wind components equated to zero. Correlations were made to rectify the influence of water vapor on the sonic temperature measurement [25], high frequency loss of signals due to equipment malfunction [26], and the effect of air density fluctuation on CO_2_ and heat fluxes [27]. Stationary test was performed in order to exclude those data when rainfalls, dew formation, power failure, or equipment failure occurred. The procedure proposed by Foken and Wichura (1996) [28] was adopted with the rejection threshold set to 30%. During integral turbulent tests, we utilized the test for the *σω*/u*, where *σω* was the 30 min standard deviation of wind speed and u* was the friction velocity. Values that varied by more than 30% compared to the reference were rejected [29]. Two criteria were then adopted to deprive the biologically impossible values of the half-hourly data, including the following: (1) only the NEE value less than 11.3 *μ*mol (CO_2_) m^−2^ s^−1^ and the NEE value higher than −22.7 *μ*mol (CO_2_) m^−2^  s^−1^ that were selected for further analysis [19] and (2) the flux under weak turbulence (friction velocity, u* < 0.15 m  s^−1^) excluded [19, 29]. After the data were processed, about 60% of the EC flux observation data remained for both the two sites were available for further analyses in this study. The mean diurnal variation (MDV) method [19] was utilized to fill short gaps (less than 2 hours) of the NEE value of the days being chosen for further analysis. The MDV utilized a 14-day moving window centered on the day of the gap, with the missing values filled with the mean fluxes within this window occurring during the same half-hourly period as the gap. The longer gaps (longer than 2 hours) were not filled in this study and the data of those days were not used.

### 2.5. Definition of Spring Tides or Neap Tides Periods of Mangrove Forests

**Figure 3:**
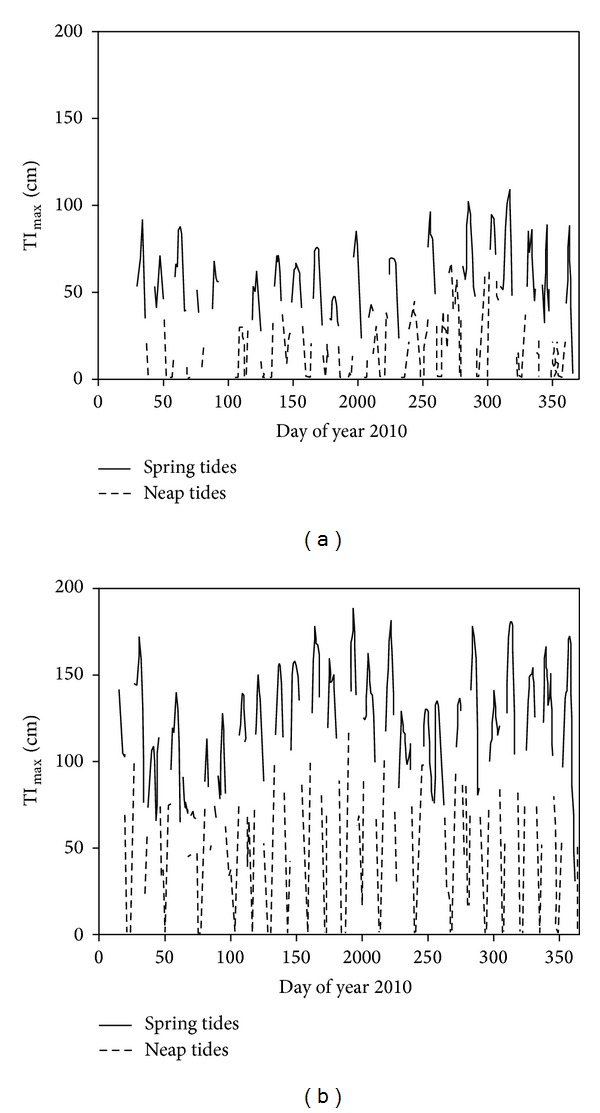
The differentiation of spring and neap tide periods of the two mangrove forest sites ((a) FJZZ and (b) GDZJ) in 2010 based on the daily maximum value of tidal inundation depth (TI_max⁡_).

The 30 min mean water inundation depths of the mangrove forests under the two eddy towers were calculated using the recorded tidal depth data. The daily maximum tidal inundation depth (TI_max⁡_) was also obtained to represent the daily tidal inundation depth of the mangrove forests where the two eddy towers are located [19]. According to the definition of spring tides and neap tides, those days with higher TI_max⁡_ than the average value of the next 14 days were defined as spring tides inundation periods; otherwise, they were regarded as neap tides inundation periods in our research (Figure 3). The NEE data of consecutive 3–5 days, respectively, under spring tides and neap tides in each month were chosen for further regression analysis [18].

### 2.6. Response of NEE to Light and Temperature

In order to compare the difference in the dependence of NEE on light or temperature under different tidal periods, the diurnal NEE data of the two sites under spring tides and neap tides were further divided into daytime NEE and nighttime NEE according to the values of solar irradiance. The relationship between daytime (when net radiation (Rn) was higher than 10 w/m^2^) NEE and PAR of the chosen days was estimated with the Landsberg model [30]:

(2) $$\begin{array}{c} \text{NEE}=P_{\max}\times \left(1-e^{\left(-\alpha \times (Q_{\text{PAR}}-I_{\text{comp}})\right)}\right),\; \end{array}$$

where *P*_max⁡_ is the maximum rate of photosynthesis (*μ*mol (CO_2_) m^−2^ s^−1^), *α* is apparent quantum yield (*μ*mol (CO_2_) per *μ*mol photon), *Q*_PAR_ is PAR (*μ*mol (Photon) m^−2^  s^−1^), and *I*_comp_ is the light compensation point (*μ*mol (Photon) m^−2^  s^−1^). The nonlinear regression analysis was performed within the SPSS statistical analysis software package to estimate the parameters related to each set of NEE and PAR measurement. The half-hourly NEE data were included in daytime when Rn was higher than 10 w/m^2^ and the NEE data were in nighttime when Rn was lower than 10 w/m^2^. The responses of daytime NEE to light were analyzed based on the regression function (2) with the range of initial value of the parameters (-100 *μ*mol (CO_2_) m^−2^ s^−1^<*P*_max⁡_<0 *μ*mol (CO_2_) m^−2^ s^−1^ , *α* > 0.001 *μ*mol  (CO_2_) per  *μ*mol  photon, *I*_comp_ > 0 *μ*mol  (photon) m^−2^ s^−1^) were obtained according to a field physiological study on the mangrove species in China [31].

For the nighttime (when Rn was lower than 10 w/m^2^) NEE, we utilized a model depicting the exponential relationship between nighttime NEE and soil temperature of 0.10 m depth [18, 32]:

(3) $$\begin{array}{c} \text{NEE}=\beta_{0}\times e^{\beta_{1}\times T_{\text{soil}}}, \end{array}$$

where *β*_0_ (*μ*mol  (CO_2_) m^−2^s^−1^) is a scaling factor and *β*_1_ is a parameter that represents the shape of the curve.

### 2.7. Statistical Analysis

The nonlinear regression analysis of NEE to PAR was performed by using the SPSS software (SPSS 15.0 for Windows, SPSS Inc., Chicago, Illinois, USA), while the nonlinear regression analysis of NEE to temperature was finished in the statistical package of SigmaPlot software (SigmaPlot 12.0 for Windows, Systat Software Inc., Chicago, Illinois, USA).

## 3. Results 

### 3.1. The Diurnal Patterns of Mangrove NEE and Its Relations to Tides

**Figure 4:**
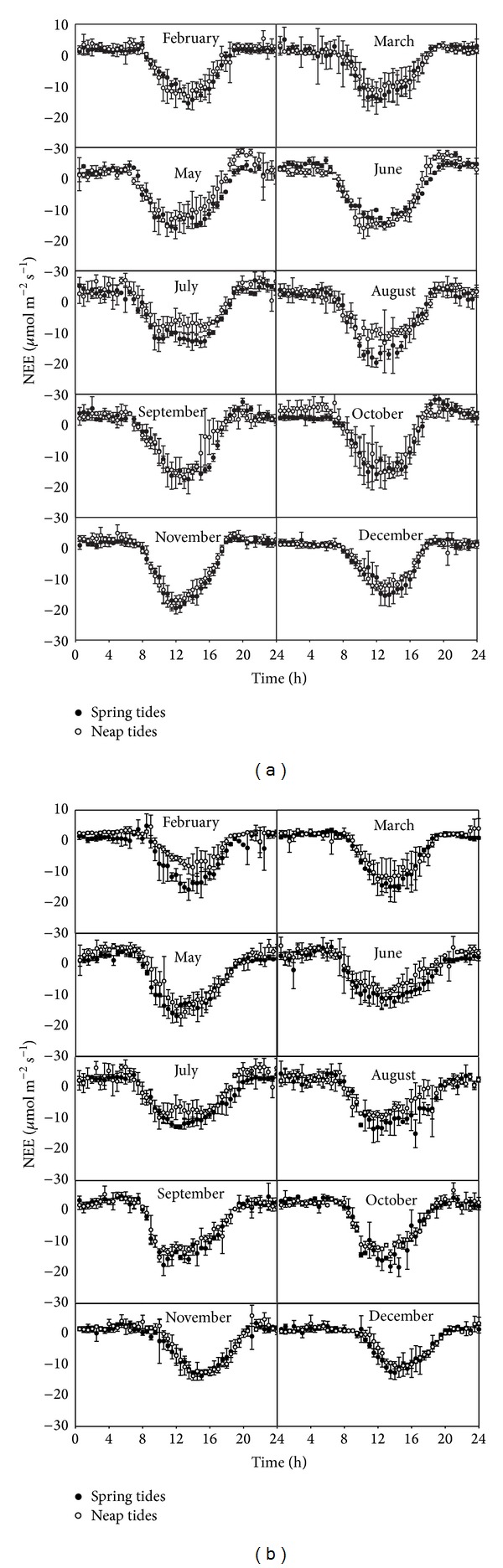
Average diurnal NEE for the selected days within spring tides or neap tides in each month of 2010 ((a) FJZZ and (b) GDZJ). The bars represent the standard deviations around the mean (*n* = 3–5).

The daytime carbon assimilation rates of mangrove forests at the two eddy tower sites reached about 10 to 20 *μ*mol · m^−2^ s^−1^ throughout the whole year with only a small increase in the mid-time of the year (May to October), while the nighttime ecosystem respiration rate increased on summer days between June and August and was higher than 4 *μ*mol · m^−2^ s^−1^ at both sites (Figure 4). The NEE data of January and April at the two sites were not showed because of missing tidal depth data due to instrument failure.

The daytime averages of NEE for the selected days during spring tide inundation periods were obviously higher than during neap tides in 7 (March, May, July, August, October, November, and December) of the 10 months of the year 2010 at the FJZZ site, while little difference was observed in the remaining 3 months (Figure 4). The same results were also observed in 8 (February, March, May, June, August, September, October, and December) of the 10 months at the GDZJ site, but no obvious differences were found in the remaining 2 months (Figure 4).

The average nighttime NEE on the days with spring tide inundation was significantly lower than those on the days with neap tides in February, October, and November of 2010 at the FJZZ site and increased in March and June, while no differences were observed in other months (Figure 4). The decreased nighttime NEE during spring tides was also observed at the GDZJ site in February, May, and June of 2010 with no or little difference in the rest of the months (Figure 4).

### 3.2. Tidal Effect on Responses of Daytime NEE to PAR

**Figure 5:**
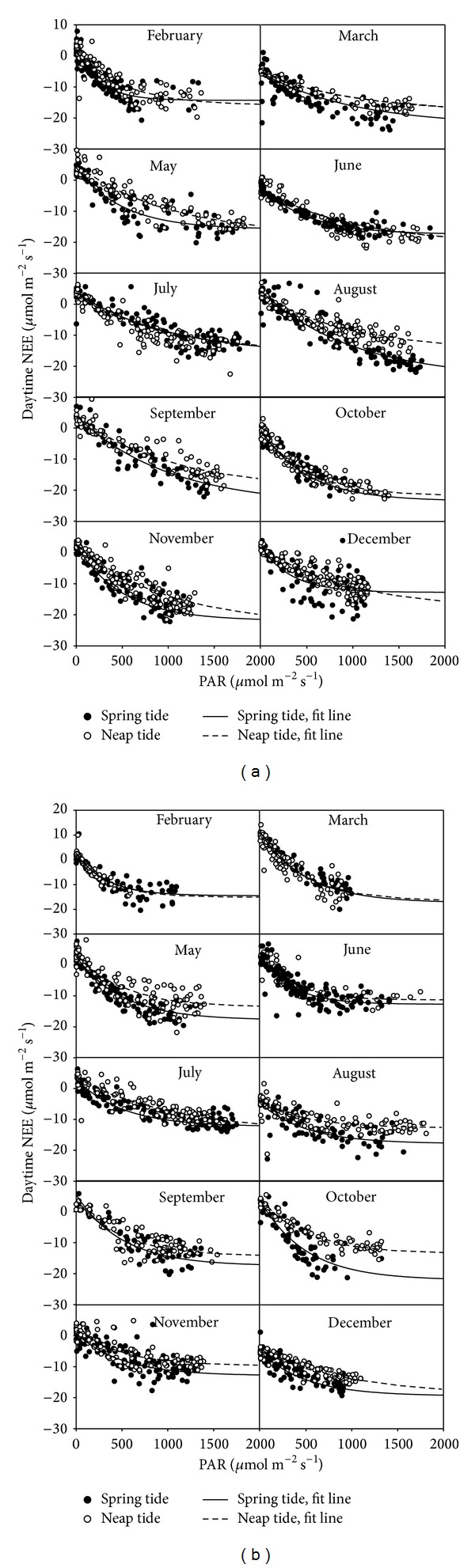
Daytime NEE versus PAR partitioned by spring tides and neap tides for the 10 selected months of the year 2010 ((a) FJZZ and (b) GDZJ). Fit (solid and dashed lines) curves from nonlinear regression analysis of daytime NEE versus PAR based on (2) (the Landsberg model) were included.

The fit curves in Figure 5 demonstrated the same pattern for the responses of NEE of mangrove forests to light with higher sensitivity during spring tide periods than neap tide period in most of the investigated months at both study sites. At the FJZZ site, the NEE-PAR response curve under spring tides was not different from neap tides in July 2010 (Figure 5). At the GDZJ site, the NEE-PAR response curves in February and March showed no difference between spring tides and neap tides (Figure 5).

The *P*_max⁡_, *α*, and *I*_comp_ values obtained from the regression analyses in each month of FJZZ and GDZJ were separately listed in Tables 1 and 2. Comparison of these parameters in each month further confirmed the patterns from our above analyses. The *P*_max⁡_ values of FJZZ during spring tides were higher than neap tides in 5 out of 10 months, and the *I*_comp_ values in 8 out of 10 months were lower during spring tides than neap tides (Table 1). At the GDZJ site, the *P*_max⁡_ values in 8 out of 10 months were higher during spring tides, while the *I*_comp_ values in 8 out of 10 months were lower during spring tides in comparison with the values for the neap tide periods (Table 2).

### 3.3. Tidal Effect on Responses of Nighttime NEE to Soil Temperature

**Figure 6:**
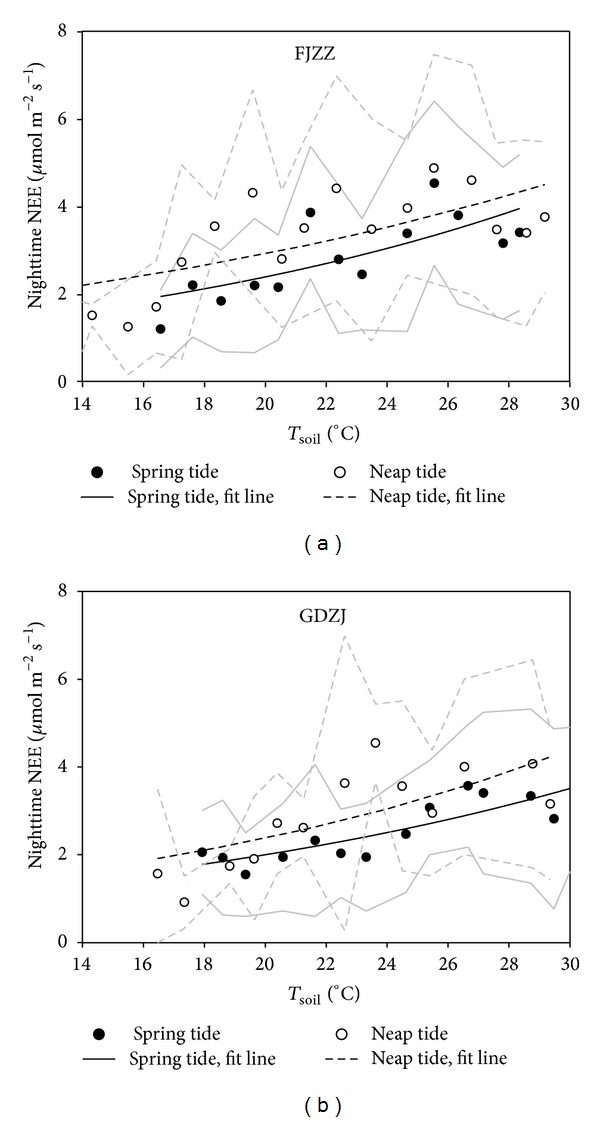
Nighttime NEE versus soil temperature at 10 cm depth partitioned by spring tides and neap tides of the year 2010 at FJZZ and GDZJ. Each subset of the data was divided into bins corresponding to the increasing soil temperature, and the averages (circles) and standard deviation (solid and dashed grey lines) were computed for each bin. Fit curves (solid and dashed black lines) from linear regression analysis of the averages of nighttime NEE versus temperature based on (3) were included.

Figure 6 showed the response curves of NEE to soil temperature based on (3) incorporating all the chosen days, respectively, during spring tides or neap tides in 2010 at both study sites, and the related parameters from these curves were reported in Table 3. The results showed inhibited nighttime NEE under spring tidal inundation in comparison with the values under neap tide inundation (Figure 6). With the *β*_0_ values for both study sites were significantly lower during spring tides than neap tides (Table 3). We also tried to generate response curves of NEE data to soil temperature for each month, but it was not doable due to little variation in the soil temperature over such short period. Thus, we pooled all 10-month data for these above analyses.

## 4. Discussions

Our results indicate that the daytime NEE values of mangrove forests in southern China are higher under spring tides than neap tides in most cases at both study sites with contrasting tidal regimes, while the decrease of nighttime NEE under spring tide inundation was observed in some months but general pattern could be obtained. However, further regression analysis using empirical models showed differential responses of NEE to light or temperature between spring tides and neap tides, with increased sensitivity of daytime NEE value to PAR but decreased sensitivity of night time NEE value to temperature under spring tide inundation. Since the increase of *P*_max⁡_ and decrease of *I*_comp_ both are good indicators of increased carbon assimilation capacity of plants [18, 30], the significantly increased *P*_max⁡_ values and decreased *I*_comp_ values obtained from the regression analysis of NEE-PAR relationship at our two mangrove sites indicated that the carbon assimilation capacity of mangrove forests was increased by spring tides in comparison with neap tides. Since the diurnal variation of daytime NEE was dominated by gross primary productivity (GPP) in forest ecosystems [33], results might indicate that not only the ecosystem respiration rate was lowered but also the GPP was increased during spring tides in relative to those during neap tides although further analysis should be performed. Thus, we hypothesize that the increased daytime NEE values under spring tide inundation should be attributed to both lowered ecosystem respiration and higher GPP during spring tide periods comparing with those during neap tides.

According to our knowledge, our study is the first one to report consistently increased daytime NEE values of mangrove forests during spring tides inundation periods throughout the year. A positive relation between tidal range and net ecosystem production was observed by Alongi and Brinkman (2011), which proved some direct support for this conclusion [34]. Although we hypothesize that this change is likely due to increased GPP during spring tides, no direct measurements of mangrove GPP are available to test this hypothesis. However, the results from a few individual level controlled experiments may provide some indirect supports for our hypothesis. For example, several greenhouse manipulation experiments on possible physiological response of mangrove seedlings to different tidal water inundations indicated that photosynthetic rate, transpiration rate, and stomatal conductance of mangrove seedlings were increased in 2–4-hour daily tidal inundations comparing with the no inundation control [10, 35]. Furthermore, the simulated semidiurnal high tides that approximated current sea level conditions for red mangrove resulted in 6–21% greater maximum photosynthetic rates than the 16 cm decreased sea level [11]. In addition, increased water use efficiency and accumulation of leaf and root mineral elements of mangrove forest in response to increased tidal inundation periods within 2–6 hours were also observed [36].

The decreased ecosystem respiration under spring tide inundation has already been observed in coastal wetlands [13, 18, 19, 37]. For example, a previous field study using EC technique in salt marshes showed that tidal inundation significantly suppressed ecosystem respiration during spring tides, while daytime carbon flux was quite variable which was attributed to the variations in the plant performances during different growth periods [18]. A recent report also stressed the importance of tidal activity on salt marsh ecosystem respiration (ER) and concluded that although temperature variation controlled the whole year fluctuation of ER, tidal activity rather than temperature became the main driving factor of ER during summer months [37]. The lowered ecosystem respiration under spring tide periods was also observed in a mangrove forest in the Everglades National Park, USA [13], consistent with our results from two mangrove forests in China with much higher latitudes. Thus, we can conclude that mangrove forest ER is reduced by spring tide inundation in comparison with neap tide inundation. The relatively lower ER under spring tides was possibly due to more anoxic soils and net tidal advection of POC, DOC, and DIC from the forest into adjacent estuary waters brought by higher tidal inundation depth with longer inundation periods [19]. More field monitoring and experimental studies are needed at our study sites to verify these mechanisms.

To quantify possible impact of environmental changes associated with sea level rise including salinity and tidal inundation on ecosystem carbon exchange in coastal wetlands, it is critical to improve our understanding of carbon cycle processes in various mangrove forests, salt marshes, and so forth. Our analyses of differential responses of mangrove NEE to light or temperature under different tidal inundation periods thus provide some valuable information for predicting future dynamics of carbon cycle processes in mangrove forests under increasing sea level rise, a definite outcome of global climate change [38]. More field flux measurements over longer periods and experimental studies are urgently needed to reveal more mechanisms for how tidal water inundation affects mangrove physiological parameters especially at ecosystem level, which is crucial for projecting mangrove carbon exchange rates under different scenarios of sea level rise [11, 13, 39].

## 5. Conclusions

Comparing differences in the NEE of mangrove forests between spring and neap tides and analysing its origins should be a complicated process especially at ecosystem level, due to multiple dimensions for CO_2_ exchanges of intertidal ecosystems. Our analysis simplified this process by considering neither the lateral exchange process nor the effect of different lengths of time by tidal inundation of the day on mangrove forest NEE. Nevertheless, the results presented here at least pointed to an important phenomenon that mangrove ecosystem NEE responds differently to light or temperature between spring and neap tides, and the differences possibly resulted in consistently decreased nighttime NEE and increased daytime NEE value of mangrove forests during spring tides inundation comparing to neap tides through the year.

## Figures and Tables

**Table 1:** Parameters and R^2^ values using the Landsberg model (see (2) in the text) fitted to the daytime net ecosystem change of CO_2_ (NEE) based on photosynthetic active radiation (PAR) in 10 months of the year 2010 for the mangrove forest of Zhangjiangkou, Yunxiao, Fujian (FJZZ).

Month	Tides	DOM∗	Parameters (±standard error, *n* = 3~5)			*R* ^2^
			*P*_max⁡_	a	*I*_ comp_	
February	Spring	1–5	−14.3 (0.9)	0.004 (0.001)	**35.6 (8.0)**	0.78
	Neap	8–11	−15.9 (0.6)	0.002 (0.000)	66.6 (13.6)	0.79

March	Spring	1–5	**−20.5 (2.4)**	0.001 (0.000)	**42.1 (20.3)**	0.74
	Neap	10–12	−15.2 (0.9)	0.001 (0.000)	63.4 (13.0)	0.94

May	Spring	16–18	−15.8 (1.0)	0.002 (0.000)	**114.2 (16.9)**	0.82
	Neap	24–26	−17.8 (1.9)	0.001 (0.000)	240.5 (21.0)	0.87

June	Spring	18–20	−14.3 (0.7)	0.002 (0.000)	**152.3 (10.5)**	0.92
	Neap	4–6	−18.31 (1.6)	0.001 (0.000)	195.6 (18.2)	0.88

July	Spring	11–14	**−16.3 (2.1) **	0.001 (0.000)	208.0 (25.2)	0.82
	Neap	28–31	−15.8 (1.3)	0.001 (0.000)	148.6 (16.2)	0.85

August	Spring	24–28	**−23.9 (2.4)**	0.001 (0.000)	**165.1 (19.9)**	0.87
	Neap	14–17	−15.1 (1.7)	0.001 (0.000)	170.8 (21.2)	0.82

September	Spring	25–27	**−24.9 (3.4)**	0.001 (0.000)	**146.6 (16.7)**	0.90
	Neap	14–16	−19.5 (3.2)	0.001 (0.000)	180.5 (15.9)	0.87

October	Spring	8–12	**−21.9 (3.9) **	0.002 (0.000)	132.3 (13.0)	0.85
	Neap	14–17	−19.9 (1.3)	0.002 (0.000)	131.9 (7.9)	0.93

November	Spring	21–25	−21.9 (2.1)	0.002 (0.000)	**51.2 (14.0)**	0.86
	Neap	14–18	−23.4 (2.5)	0.001 (0.000)	92.1 (14.5)	0.89

December	Spring	5–9	−12.8 (1.1)	0.003 (0.001)	**44.5 (22.0)**	0.57
	Neap	17–21	−18.4 (3.9)	0.001 (0.000)	83.6 (20.1)	0.82

*DOM: day of the month.

The numbers in bold indicated higher *P*_max⁡_ or lower *I*_comp_ during spring tides than neap tides.

**Table 2:** Parameters and *R* ^2^ values using the Landsberg model (see (2) in the text) fitted to the daytime net ecosystem change of CO_2_ (NEE) based on photosynthetic active radiation (PAR) in 10 months of the year 2010 for the mangrove forest of Zhanjiang, Guangdong (GDZJ).

Month	Tides	DOM∗	Parameters (±standard error, *n* = 3~5)			*R* ^2^
			*P*_max⁡_	a	*I*_ comp_	
February	Spring	7–9	−14.5 (1.0)	0.003 (0.001)	**51.6 (11.2) **	0.82
	Neap	3–5	−15.1 (1.4)	0.003 (0.000)	57.6 (4.2)	0.92

March	Spring	22–24	**−19.9 (1.8) **	0.002 (0.000)	69.5 (8.3)	0.94
	Neap	15–18	−19.4 (1.7)	0.002 (0.000)	46.1 (9.0)	0.87

May	Spring	1–4	**−17.8 (0.9)**	0.002 (0.000)	**71.2 (8.6) **	0.90
	Neap	6–9	−13.6 (0.9)	0.002 (0.000)	106.2 (13.3)	0.81

June	Spring	13–17	**−12.7 (0.7) **	0.003 (0.000)	**79.1 (12.9) **	0.74
	Neap	21–23	−11.3 (0.8)	0.003 (0.000)	95.6 (11.1)	0.80

July	Spring	9–13	−12.3 (0.6)	0.002 (0.000)	**111.9 (14.0)**	0.86
	Neap	3–6	−13.8 (2.6)	0.001 (0.000)	230.9 (28.1)	0.76

August	Spring	6–9	**−14.8 (2.2)**	0.002 (0.001)	**49.8 (37.5)**	0.57
	Neap	11–14	−8.4 (0.8)	0.002 (0.001)	98.7 (28.4)	0.56

September	Spring	28–30	**−17.5 (2.3)**	0.002 (0.000)	136.8 (27.5)	0.76
	Neap	25–27	−14.4 (1.5)	0.002 (0.000)	111.0 (22.2)	0.77

October	Spring	16–18	**−22.1 (3.4)**	0.002 (0.000)	**76.6 (9.8)**	0.81
	Neap	19–21	−13.5 (0.9)	0.002 (0.000)	126.7 (12.9)	0.91

November	Spring	6–10	**−12.9 (1.8)**	0.002 (0.000)	**29.0 (31.1)**	0.63
	Neap	1–4	−9.7 (0.9)	0.002 (0.001)	92.7 (22.1)	0.67

December	Spring	21–24	**−16.8 (3.3) **	0.002 (0.001)	**32.0 (19.7)**	0.74
	Neap	26–29	−16.5 (4.2)	0.001 (0.000)	76.3 (20.5)	0.82

*DOM: day of the month.

The numbers in bold indicated higher *P*_max⁡_ or lower *I*_comp_ during spring tides than neap tides.

**Table 3:** Parameters and *R* ^2^ values for the average nighttime NEE fitted to soil temperature (see (3) in the text) on the chosen spring and neap days of the year 2010 at two mangrove study sites.

Site	Tides	Parameters (±standard error)		*R* ^2^	*P*
		*β*_0_	*β*_1_		
FJZZ	Spring	0.66 (0.08)	0.06 (0.01)	0.14	<0.0001
	Neap	0.91 (0.11)	0.05 (0.01)	0.16	<0.0001

GDZJ	Spring	0.67 (0.10)	0.05 (0.01)	0.10	<0.0001
	Neap	0.96 (0.13)	0.05 (0.01)	0.10	<0.0001
